# Predisposing Factors and Outcome of Malpresentations in an Institute

**Published:** 2018-06-30

**Authors:** Smrity Maskey, Yam Dwa

**Affiliations:** 1 Department of Obstetrics and Gynaecology, KIST Medical College and Teaching Hospital, Lalitpur, Nepal

**Keywords:** *malpresentations*, *maternal and fetal outcome*, *predisposing factors*

## Abstract

**Introduction:**

This study was done to find out the incidence of malpresentation among all deliveries with various types of Malpresentations, its mode of delivery, maternal and fetal predisposing factors with outcome.

**Methods:**

This was a cross sectional descriptive study done at KIST Medical College and Teaching Hospital. Review cases of women admitted in labor after 22 weeks with malpresentation was done. Maternal/fetal predisposing factors were recorded.

**Results:**

Total delivery in study period was 4009 where 101 (2.5%) were of malpresentation. Breech was the commonest malpresentation 83 (82.1%). Assisted vaginal delivery occurred in 16 (15.8%) and 953 (84.2%) caesarian section. Malpresentations was common in primigravida 62 (61.3%). Half (47.2%) cases had one/more predisposing factors, commonest being oligohydramnious 7 (6.9%). Out of 108 babies with malpresentation, 10 had perinatal deaths and 10 had NICU admissions. Congenital anomaly was found in 4 babies.

**Conclusions:**

The most common type of malpresentation was breech common in primigravida with oligohydramnios as contributing factor.

## INTRODUCTION

Malposition is abnormal positions of fetal vertex in relation to maternal pelvis. At onset of labor, the incidence is about 10% of all vertex presentation.^1^ Malpresentation is presentation other than vertex. Breech presentation being the commonest (3%-4%) at term but more common in earlier gestations.^2^ Many studies were conducted to find the cause of malpresentation and its maternal / fetal outcome^3–5^ focused on gravida, malpresentation,^4^ and association with their route of delivery.^3–5^ Malpresentations usually ends increasing operative delivery,^6^ leading to increased adverse outcome for mother and baby. The incidence of cord prolapse is 5-10% in transverse lie, 3% in breech (flexed leg)^7^ and 10% in compound presentation.

Taking prompt action may save life of mother and baby if the delivery becomes obstructed due to abnormal presentation.^7^ The risk of cord prolapse is 1 in 300 deliveries which decreases with elective caeserian section.^8^ Malpresentation is quite common in multiple pregnancies (10%).^9^

This study aims in finding the commonest type of malpresentation, its diagnosis with prompt management and outcome.

## METHODS

A cross sectional descriptive study was conducted in KIST medical college from December 2013 to February 2014. Ethical approval was taken. Data were collected from medical record of all the pregnant women between June 2008 to January 2014. All delivery with malpresentation and malposition that occurred during the study period was included. On the other hand those gestation before after age of viability (22 weeks) with diagnosis of malpresentation were not included in the study. All parameters like age, gravida, parity, gestation age, ANC visits, type of malpresentation, mode of delivery, baby's birth weight, Apgar score, neonatal outcome, maternal complications were noted. Similarly any maternal or fetal predisposing factors were also looked for and recorded. Descriptive analysis were done using Microsoft Excel.

## RESULTS

**Figure 1.**
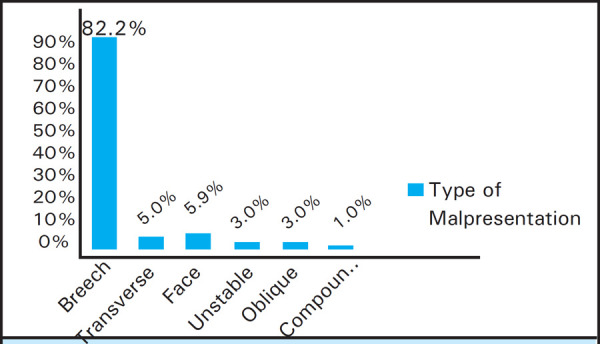
Types of Malpresentation.

The total number of delivery in the study period of five and half years was 4009. Among them 101 (2.5%) cases were of malpresentation. Breech was the most common malpresentation 83 (82.1%) among these malpresentations (Figure 1).

**Figure 2.**
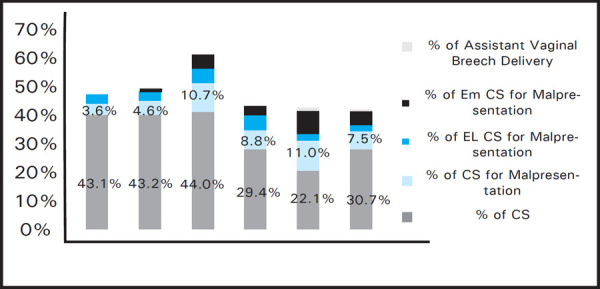
Modes of delivery in Malpresentation.

Out of these cases of malpresentation, assisted vaginal delivery occurred in 16 (15.8%) and 953 (84.2%) underwent caesarian section (Figure 2).

The mean age of these women were 23.4 years. Malpresentation was more common in primigravida 62 (61.3%) as compared to multigravida (Table 1). Most of the malpresentations were diagnosed at ANC before the onset of labor; only 5 (4.9%) had no ANC and were diagnosed as malpresentation at labor. About half (47.2%) of the cases had one or more associated predisposing factors for malpresentation. The most common associated factor mentioned was Oligohydramnious 7 (6.9%) followed by IUGR, twin, IUFD, APH, Cord prolapse, abnormality and previous section. None of the cases had uterine anomalies documented though it is a common associated factor. Only 2 (1.9%) of cases had placenta previa.

**Table 1.** Malpresentation with complications.

Year	Malpres entation only	Malpresentation with complication											Total
		Oligohydr amnious	Decrease FM	IUGR	Twin	Pre CS	IUFD	APH	Previa	PIH	Cord Prolapse	Abnor mality	
2065	2	0	0	0	0	0	0	0	0	0	0	0	2
2066	3	1	1	0	0	0	0	0	0	0	0	0	5
2067	17	2	0	2	2	0	0	0	1	0	0	0	24
2068	13	2	0	0	2	1	0	0	0	0	0	0	18
2069	18	1	1	0	3	4	2	1	0	0	0	3	33
2070	9	1	0	0	0	0	3	0	1	2	2	1	19
Total	62	7	2	2	7	5	5	1	2	2	2	4	101
n (%)	61.39%	6.93%	1.98%	10.53%	6.93%	4.95%	4.95%	0.99%	1.98%	1.98%	1.98%	3.96%	100.00%

Total 108 babies were born including seven sets of twins. Among these babies 10 had perinatal deaths (5 IUFD and 5 early Neonatal Deaths), 10 babies had NICU admissions for birth asphyxia. Low birth weight (less than 2.5 kg) baby's rate was 40.74%. Congenital anomaly was found in 4 babies.

## DISCUSSION

Malpresentation and malposition is commonly encountered obstetric problem which accounts of 3-4%. In this study in a span of five and half years its incidence was 2.5%.

Breech was the most common malpresentation in our hospital study accounting for 82.1% which is consistent to the study from U.K.^1^ in which breech as malpresentation was 85%. In present study the most common mode of delivery was caesarian section i.e. 84.2%. Assisted vaginal delivery accounted for 15.8% which was seen only in breech presentation. The incidence of breech vaginal delivery is low than a CS rate which corresponds with the study of Nordin NM.^10^ This also supports the study of reference.^10–12^ For most of the examined parameters statistically significant differences were found in mortality and morbidity between the vaginally delivered group and the caesarean section group in three birth weight categories which corresponds to the study of de Leeuw JP.4

In the study malpresentation with complication was the most common cause that increased caesarian rate. The commonest causes were twin and oligohydramnious 7 ( 6.9%) each. Because we include only studies that compared non-cephalic presenting twins with each other, these reports were excluded which is supported by the reverence.^9^ A limitation of this study was no uterine anomaly was detected despite it is the most common cause of malpresentation. In the study cord prolapsed was seen in 2 cases (2%) out of 101 malpresentation cases which is very high in contrast to study of Kouam L where 39 ( 0.1%) cases of cord prolapsed were found out of 20,000 cases. This disparity can be because of the sample size.

Due to good ANC, 95.1% patient were diagnosed with malpresentation antenatally and were managed electively in contrast to 4.9% patient who had no ANC. This indicates that good ANC can detect malpresentation which will have good prognosis for mother and baby. Malpresentation was more common in primi gravida which was 61.3%. This might indicate that the patient coming to hospital for deliveries were mostly primi.

NICU admissions were 5 patients for birth asphyxia and were seen in emergency LSCS patients only. Elective LSCS had good neonatal outcome. Among the 3 category of baby birth weight in the study very low birth weight was found only in pre-term pregnancy whereas it was more than 2.5kg in term and post term pregnancies.

## CONCLUSIONS

Malpresentation accounted for 2.5% in the study with breech as the most common type. Most malpresentations were diagnosed at ANC through clinical finding and ultrasonography while only 4.9% had no ANC and diagnosed at labor. The most common associated factor of malpresentation was oligohydramnios and twins. The most common mode of delivery was Caesarian section. In caesarian section emergency section was more compared to elective section due to associated complications and late detection of malpresentation. The maternal and fetal outcome was good with no maternal mortality and 10 perinatal morbidity. Anencephaly was the only congenital abnormality encountered in the study (4 cases). Future research about this subject might be useful.
